# ‘Stay at home …’: exploring the impact of the COVID-19 public health response on sexual behaviour and health service use among men who have sex with men: findings from a large online survey in the UK

**DOI:** 10.1136/sextrans-2021-055039

**Published:** 2021-09-20

**Authors:** Alison R Howarth, John Saunders, David Reid, Isabelle Kelly, Sonali Wayal, Peter Weatherburn, Gwenda Hughes, Catherine H Mercer

**Affiliations:** 1 UCL Institute for Global Health, University College London, London, UK; 2 The National Institute for Health Research Health Protection Research Unit in Blood Borne and Sexually Transmitted Infections at University College London in partnership with Public Health England, London, UK; 3 Blood Safety, Hepatitis, STIs and HIV Division, National Infection Service, Public Health England, London, UK; 4 Sigma Research, Public Health, Environments and Society, London School of Hygiene & Tropical Medicine, London, UK

**Keywords:** sexual behavior, COVID-19, sexual health, population surveillance, homosexuality, male

## Abstract

**Objectives:**

The first UK national lockdown began on 23 March 2020, in response to the COVID-19 pandemic, and led to reduced STI/HIV service provision in the UK. We investigated sexual behaviour, use and need for sexual healthcare during the pandemic.

**Methods:**

Participants (N=2018), including men (cis/transgender), transwomen and gender-diverse people reporting sex with another man (cis/transgender) or non-binary person assigned male at birth, completed an online cross-sectional survey (23 June 2020–14 July 2020), in response to adverts on social media and dating apps.

Sexual behaviour, service use and unmet need for STI testing (any new male and/or multiple condomless anal sex (CAS) partners without STI testing) in the 3 months since lockdown began were examined and compared using multivariable analyses with an equivalent 3-month period in a 2017 survey (N=1918), conducted by the same research team.

**Results:**

Since lockdown began, 36.7% of participants reported one or more new partners, 17.3% reported CAS with multiple partners, 29.7% HIV testing (among 1815 of unknown/negative status), 24.9% STI testing and 15.4% using pre-exposure prophylaxis (PrEP).

Since lockdown began, 25.3% of participants had unmet need for STI testing. This was more likely among Asian versus white participants (adjusted OR (aOR)=1.76, (1.14 to 2.72), p=0.01); for participants living in Scotland (aOR=2.02, (1.40 to 2.91), p<0.001) or Northern Ireland (aOR=1.93, (1.02–3.63), p=0.04) versus England; and for those living with HIV (aOR=1.83, (1.32 to 2.53), p<0.001).

Compared to 2017, the equivalent 2020 subsample were less likely to report new male partners (46.8% vs 71.1%, p<0.001), multiple CAS partners (20.3% vs 30.8%, p<0.001) and have unmet need for STI testing (32.8% vs 42.5%, p<0.001) in the past 3 months.

**Conclusions:**

We found potential for ongoing STI/HIV transmission among men who have sex with men during the initial UK lockdown, despite reduced sexual activity, and inequalities in service access. These findings will support public health planning to mitigate health risks during and after the COVID-19 response.

## Introduction

On 11 March 2020, the WHO declared COVID-19 a pandemic.^1^ In response, the UK government began the UK’s first national lockdown on 23 March, under the slogan, ‘stay at home, protect the NHS, save lives’.^2^ People were required to stay at home (except for very limited purposes), certain businesses and venues were closed, and public gatherings of more than two people were banned. An extensive reduction in sexual health service (SHS) provision was recommended, including pauses in routine vaccination and asymptomatic testing for STIs and HIV.^3 4^

Men who have sex with men (MSM) experience a disproportionate burden of bloodborne and sexually transmitted infections (BBSTIs)^5 6^ but social distancing (avoiding close contact with anyone from a different household) may reduce sexual contacts and decrease BBSTI transmission. In online surveys of MSM in the UK, 24% of a community sample (April–May 2020) reported some casual sex during lockdown,^7^ and 76% of a clinic sample (August 2020) reported sex during lockdown, of whom 76% reported sex with partners outside their household.^8^

It remains unclear whether reduced service provision after lockdown resulted in unmet need for sexual healthcare in the UK. One central London SHS reported 80% fewer prescriptions for HIV post-exposure prophylaxis (PEP) *following* compared with an equivalent period *before* lockdown,^9^ while a community survey found that two-thirds of MSM on HIV pre-exposure prophylaxis (PrEP) had paused or discontinued use during lockdown.^7^ These findings suggest reduced sexual activity and/or reduced access to SHS.

We explored changes in sexual behaviour and use of SHS among cisgender men, transgender and gender-diverse people who have sex with men in the UK, during the UK COVID-19 response to understand service need. The paucity of evidence on sexual risk behaviours among transgender and gender-diverse people in the UK^10–12^ underlines the importance of identifying the sexual health needs of these groups.^13^

Previous research demonstrated the feasibility and acceptability of rapid risk assessment methods to enhance existing surveillance measures in response to public health incidents and outbreaks.^14 15^ We present findings from our community-based cross-sectional online survey, using this approach, including a comparison with an equivalent survey conducted as part of an ongoing programme of research (online supplemental appendix 1) by the same team in 2017.

10.1136/sextrans-2021-055039.supp1 Supplementary data

## Methods

### Study design

This is a cross-sectional online community survey.

### Setting and sampling

#### 2020 survey

Participants were recruited from social networking sites (Facebook, Twitter, Instagram) and geospatial dating applications (apps) (Grindr, Hornet) between 23 June and 14 July 2020. An advert on these sites and apps directed individuals to the online survey. Eligible individuals were as follows: UK residents; aged 16 years or older; men (cisgender or transgender), transwomen, or gender-diverse and assigned male at birth; who reported sex since the beginning of 2019 (equivalent of the past 18 months) with a man (cisgender or transgender) or non-binary person assigned male at birth. Online consent was obtained from all participants: information about the voluntary and anonymous nature of the survey, its content and the research team were provided at the beginning of the questionnaire, and participants ticked a box to confirm that they had read the information and agreed to participate.

#### Comparison with the 2017 survey

Data from our equivalent 2017 online survey of MSM were included in comparative analyses.^14 15^ We describe this previous survey as the ‘2017 survey’ as distinct from the ‘2020 survey’. The 2017 survey collected data between March and May 2017 and included UK-resident cisgender and transgender MSM aged 16 years or older, who reported sex with a man in the past 12 months. Participants were recruited via social networking/dating platforms. Our comparative analyses of the 2017 and 2020 surveys include only cisgender men recruited via the dating platform Grindr, to maximise sample equivalence.

### Data collection

Both surveys used anonymous self-completion online questionnaires taking about 10 min to complete. No financial incentive was offered. The 2020 questionnaire was based on the 2017 questionnaire and included sections on HIV/STI testing, vaccination, PrEP use, STI symptoms, use of SHS, sexual relationships and behaviour, and drug use. In the 2020 survey, questions about the last occurrence of risk behaviour and service use included ‘lookback periods’ which centred around the start of the first UK national lockdown (*since lockdown (23 March 2020), between mid-December 2019 and lockdown, between January 2019 and mid-December 2019, before 2019*).

The lookback periods for the 2017 survey included the past 3 months. Both surveys therefore included the past 3 months as a key reference period for questions about behaviour and service use, as this equated to the time since the national lockdown for the 2020 survey (online supplemental appendix 2 shows the timing of the 3-month lookback periods). This 3-month period is used for the comparisons between the 2020 and 2017 surveys.

### Data analysis

The χ^2^ test was used to examine differences in proportions for comparisons within the 2020 survey sample and between the 2020 and 2017 survey samples, and the Mann-Whitney U test for comparisons involving continuous variables.

Informed by national guidelines for 3 monthly STI testing among men reporting STI risk behaviours,^16^ we created an indicator of ‘unmet need for STI testing’, defined as reporting one or more new male partners and/or multiple condomless anal sex (CAS) partners in the past 3 months without testing for STIs over the same period.

Binary logistic regression was used to (1) examine associations between explanatory variables and the indicator of unmet need for STI testing; and (2) examine whether survey sample (2017 vs 2020) remained a significant predictor of sexual behaviour and unmet need for STI testing when other explanatory variables were included in the model. Crude (OR) and adjusted odds ratios (aOR) are presented, with 95% CIs.

## Results

### Risk behaviour and health service use from the 2020 survey

In total, 2018 participants took part in the 2020 survey. The majority (97.0%) were cisgender men, but 18 transmen, 10 transwomen, 1 man assigned intersex at birth and 31 gender-diverse people assigned male at birth also participated.

Recruitment was evenly split between social networking sites (47.1%) and dating apps (52.9%). Their median age was 40 years (IQR 29–52 years; range 16–77 years). The majority identified as white (88.3%), were resident in England (86.4%) and were born in the UK (78.1%). More than half had a degree (58.2%) and 58.1% were currently employed. A fifth (20.3%) were on furlough (whereby the government paid 80% of salaries for people unable to work due to COVID-19 restrictions), reduced hours or had been made redundant since lockdown began. One-third of participants were living alone (35.8%), one-third were living with a partner(s) (30.4%) and 59.1% were single. One in 10 participants was living with HIV (10.1%).

Participants recruited via dating apps were more likely to be from a minority ethnic group (14.0% vs 9.3%, p=0.001), single (71.5% vs 45.1%, p<0.001) and living alone (39.0% vs 32.1%, p=0.001), and less likely to be living with partner(s) (21.7% vs 40.2%, p<0.001) compared with those recruited through social networking sites (online supplemental appendix 3).

#### Sexual and risk behaviours since lockdown began

**Figure 1:**
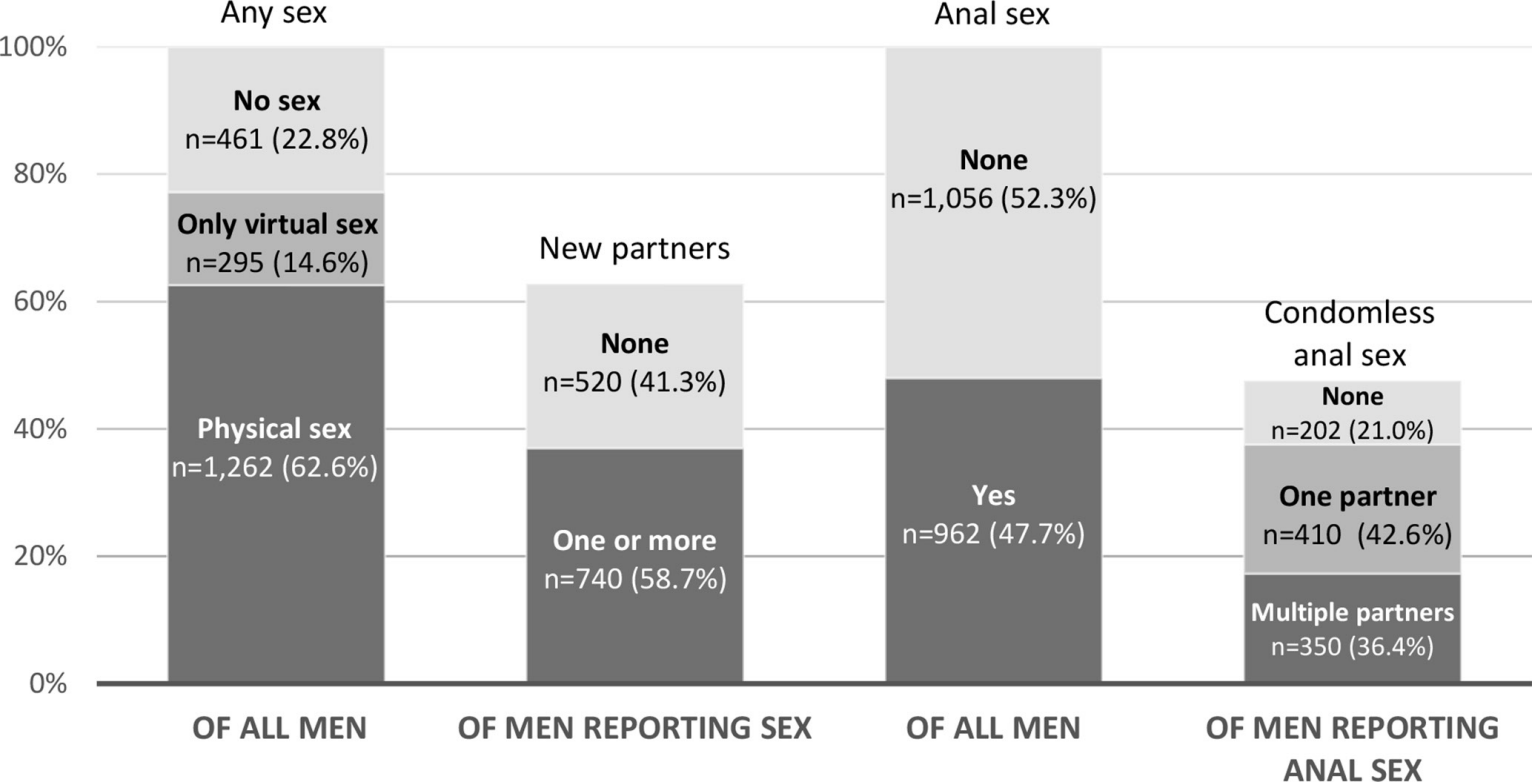
Reported sexual activity with men since lockdown.

More than half of participants reported physical sex with a man (defined as ‘any activity intended to achieve orgasm (or close to orgasm) for one or both partners’) (62.6%) (figure 1) and 14.6% reported only virtual sex (defined as ‘sex or sexting’) since lockdown began. Among participants reporting physical sex since lockdown began, 58.7% reported one or more new partners (36.7% of all participants). Among those reporting one or more new partners, most had met partners through dating apps (80.6%), 29.5% through websites and 17.4% through cruising locations (defined as ‘street, roadside service area, park, beach, lavatory’). Half of all participants reported anal sex with a man since lockdown began, and among this group, 42.6% reported CAS with one partner and 36.4% with multiple partners (20.3% and 17.3% of all participants, respectively). Use of chemsex drugs (crystal methamphetamine, mephedrone, gamma-hydroxybutyrate/gamma-butyrolactone) was reported by 13.0% and 3.8% of participants, ever and since lockdown began, respectively. Fewer reported injecting drug use: 3.0% and 0.8%, ever and since lockdown began, respectively.

#### STI testing since lockdown began

Among all participants, 81.5% had ever tested for STIs with 24.9% testing since lockdown began. Of those testing since lockdown began, 17.7% had tested positive (4.4% of all participants), 78.1% had tested negative and 4.2% were still waiting for results. Free online self-sampling services were the most commonly used route for STI testing since lockdown began (17.3% of participants; 69.6% of lockdown testers), followed by visiting an SHS (7.4% of participants; 29.6% of lockdown testers). Most participants had not experienced any STI symptoms since lockdown began (85.7%).

#### HIV testing before and after lockdown began

Among people with unknown or negative HIV status (n=1815), 84.5% had ever had an HIV test, 22.9% had last tested ‘just before’ lockdown (in the 3 months between December 2019 and lockdown) and 29.7% had last tested in 3 months since lockdown began (table 1). Those who had last tested since lockdown began were less likely to have done so at a sexual health clinic (22.3% vs 70.2%) and more likely to have used a free online self-sampling service (64.3% vs 17.1%) or a self-testing kit (7.8% vs 3.4%, p<0.001) compared with those testing just before lockdown.

**Table 1:** Comparison of last visit to the sexual health clinic, last HIV test and last use of PrEP in the 3 months before and since lockdown

	In 3 months before lockdown began n (%)	In 3 months since lockdown began n (%)	P value
Testing for HIV*			
When last tested for HIV (n=2018)	415 (22.9)	539 (29.7)	
Where last tested for HIV (n=948)			<0.001
Sexual health clinic	288 (70.2)	120 (22.3)	
Free online self-sampling	70 (17.1)	346 (64.3)	
Self-testing kit	14 (3.4)	42 (7.8)	
Other	38 (9.3)	30 (5.6)	
**Visiting the sexual health clinic**			
When last visited sexual health clinic (n=2018)	487 (24.1)	229 (11.3)	
Reasons for last visit to sexual health clinic† (n=716)			
Sexual health check-up	306 (62.8)	68 (29.7)	<0.001
To get PEP or PrEP	132 (27.1)	81 (35.4)	0.02
Had symptoms	40 (8.2)	48 (21.0)	<0.001
Worried might have STI	65 (13.3)	19 (8.3)	0.05
Needed vaccination	40 (8.2)	18 (7.9)	0.87
Partner diagnosed with STI	31 (6.4)	17 (7.4)	0.60
Treatment after positive test	18 (3.7)	22 (9.6)	0.001
Check-up after positive test	20 (4.1)	12 (5.2)	0.49
Follow-up after online test	8 (1.6)	18 (7.9)	<0.001
Partner had symptoms	15 (3.1)	6 (2.6)	0.73
Could not get online test	8 (1.6)	1 (0.4)	0.18
**Using PrEP‡**			
When last used PrEP (n=470)	102 (21.7)	309 (65.7)	
Whether had new male partners since lockdown began (n=410)			<0.001
No new partners	81 (80.2)	112 (36.2)	
One or more new partners	20 (19.8)	197 (63.8)	

Table created by coauthors.

*Among those with negative or unknown HIV status.

†Participants tick all reasons that apply.

‡Among those who have ever used PrEP.

PEP, post-exposure prophylaxis; PrEP, pre-exposure prophylaxis.

#### Sexual health clinic visits before and after lockdown began

Among all participants, 81.0% had ever visited a sexual health clinic, 24.1% just before lockdown and 11.3% since lockdown began. Table 1 lists reasons given for last visiting a sexual health clinic. Compared with those last visiting just before lockdown, those last visiting since lockdown began were more likely to attend with symptoms (21.0% vs 8.2%, p<0.001), for treatment after a positive test (9.6% vs 3.7%, p=0.001) or for follow-up after an online test (7.9% vs 1.6%, p<0.001). They were less likely to attend for a sexual health check-up (29.7% vs 62.8%, p<0.001).

#### PrEP use before and after lockdown began

Among all participants, 23.4% had ever used PrEP, 5.1% last used it just before lockdown and 15.4% last used PrEP since lockdown began. Among PrEP users (those who reported ever using PrEP), 21.7% had last used PrEP just before lockdown and 65.7% since lockdown began. Those who had last used PrEP since lockdown began were more likely to report one or more new partners since lockdown began (63.8% vs 19.8%), whereas those who had last used PrEP just before lockdown were more likely to report no new partners since lockdown began (80.2% vs 36.2%, p<0.001).

#### Unmet need for STI testing since lockdown began

Among all participants, 39.8% reported multiple CAS partners and/or one or more new male partners since lockdown began. Among these, 36.5% had tested for STIs since lockdown began, while 63.5% had not, indicating unmet need for STI testing (25.3% of the entire sample) (table 2). Participants recruited via dating apps were more likely to have this unmet need than those recruited via social media (aOR=2.02, (1.62 to 2.53), p<0.001), as were Asian compared with white participants (aOR=1.76, (1.14 to 2.72), p=0.01), those living in Scotland (aOR=2.02, (1.40 to 2.91), p<0.001) or Northern Ireland (aOR=1.93, (1.02 to 3.63), p=0.04) compared with England and those living with HIV (aOR=1.83, (1.32 to 2.53), p<0.001). Unmet need was less common among participants aged 60 years and older compared with 16–29 year olds (aOR=0.60, (0.37 to 0.96), p=0.04). There was no difference between the English regions on unmet need (online supplemental appendix 4).

**Table 2:** Unmet need for STI testing† since lockdown began, by recruitment site and background characteristics

	n (%)	Unadjusted OR (95% CI)	P value	aOR (95% CI)*	P value
Recruitment site					
Social media	169 (17.8)	1		1	
Dating apps	341 (31.9)	2.17 (1.76 to 2.68)	<0.001	2.02 (1.62 to 2.53)	<0.001
Gender					
Cisgender male	501 (25.6)	1		1	
Other gender	9 (15.0)	0.51 (0.25 to 1.05)	0.07	0.48 (0.22 to 1.03)	0.06
Age group (in years)					
16–29	124 (24.5)	1	0.006	1	
30–44	191 (27.2)	1.15 (0.88 to 1.49)	0.30	1.07 (0.81 to 1.42)	0.61
45–59	168 (26.8)	1.13 (0.86 to 1.48)	0.37	1.14 (0.85 to 1.53)	0.38
60+	27 (14.8)	0.53 (0.34 to 0.84)	0.007	0.60 (0.37 to 0.96)	0.04
Ethnic group					
White	436 (24.5)	1	0.03	1	
Black	12 (36.4)	1.76 (0.86 to 3.61)	0.12	1.53 (0.73 to 3.21)	0.27
Asian	39 (35.8)	1.72 (1.15 to 2.58)	0.009	1.76 (1.14 to 2.72)	0.01
Mixed/other	23 (24.2)	0.99 (0.61 to 1.60)	0.95	0.99 (0.60 to 1.65)	0.98
Country of residence					
England	418 (24.0)	1	0.001	1	
Scotland	54 (37.5)	1.90 (1.34 to 2.71)	<0.001	2.02 (1.40 to 2.91)	<0.001
Wales	21 (24.7)	1.04 (0.63 to 1.73)	0.88	1.20 (0.71 to 2.01)	0.50
Northern Ireland	17 (37.8)	1.93 (1.04 to 3.55)	0.04	1.93 (1.02 to 3.63)	0.04
Born in the UK					
No	118 (26.8)	1		1	
Yes	392 (24.9)	0.91 (0.71 to 1.15)	0.42	0.96 (0.74 to 1.24)	0.74
Highest qualification					
Below degree	217 (25.7)	1		1	
Degree or higher	293 (25.0)	0.96 (0.78 to 1.18)	0.69	0.95 (0.76 to 1.17)	0.61
On furlough, reduced hours, redundancy					
No	392 (24.5)	1		1	
Yes	114 (28.0)	1.20 (0.94 to 1.53)	0.15	1.16 (0.90 to 1.50)	0.26
Currently single					
No	182 (22.0)	1		1	
Yes	328 (27.5)	1.34 (1.09 to 1.65)	0.005	1.09 (0.87 to 1.37)	0.44
HIV status					
Negative or unknown	439 (24.2)	1		1	
Positive	71 (35.0)	1.69 (1.24 to 2.29)	0.001	1.83 (1.32 to 2.53)	<0.001

Table created by coauthors.

*Adjusting for all variables listed in the table.

†One or more new sex partners and/or multiple condomless anal sex partners, and no STI testing.

aOR, adjusted OR.

##### Failing to get an STI test

Among participants who had not tested for STIs since lockdown began, 10.0% had tried (and failed) to get tested (7.5% of all participants). This was more commonly reported by those recruited via dating apps than via social media (8.8% vs 6.1%, p=0.02), those who identified as black (21.2%) compared with white (7.1%), Asian (11.0%) or mixed/other (6.3%, p=0.01), and by those residing in Northern Ireland (17.8%) compared with England (7.1%), Scotland (7.6%) or Wales (10.6%, p=0.04).

### Comparative analysis with the 2017 survey

Cisgender men recruited via Grindr were included in this comparison of sexual behaviour and unmet need for STI testing for the 2017 (n=1918) and 2020 (n=956) surveys. Compared to 2017, the 2020 participants were older (median age 40 years (IQR 30–50) vs 37 years (IQR 28–48), p<0.001), less likely to be of white ethnicity (86.2% vs 89.5%, p=0.02) and live in England (84.4% vs 93.7%, p<0.001). They were more likely to be single (70.9% vs 62.3%, p<0.001) and have a degree (58.6% vs 50.7%, p<0.001) (online supplemental appendix 5).

We compared sexual behaviour reported since lockdown began with that reported over an equivalent 3-month period in 2017 (table 3). Adjusting for variables associated with the survey sample, participants in 2020 were less likely to report new male partners (aOR=0.31, (0.26 to 0.37), p<0.001), anal sex (aOR=0.30, (0.25 to 0.36), p<0.001), CAS (aOR=0.48,(0.40 to 0.56), p<0.001) and multiple CAS partners (aOR=0.55, (0.45 to 0.67), p<0.001).

**Table 3:** Sexual behaviour and unmet need for STI testing in the past 3 months among cisgender MSM, by data collection period (2017 (reference category) vs 2020)

	2017 survey n (%)	2020 survey n (%)	OR (95% CI) for the 2020 survey	P value	aOR (95% CI)* for the 2020 survey	P value
Any new male partners	1127 (71.1)	447 (46.8)	0.36 (0.30 to 0.42)	<0.001	0.31 (0.26 to 0.37)	<0.001
Anal sex	1450 (76.0)	472 (49.4)	0.31 (0.26 to 0.36)	<0.001	0.30 (0.25 to 0.36)	<0.001
CAS	1036 (55.7)	250 (36.6)	0.46 (0.39 to 0.54)	<0.001	0.48 (0.40 to 0.56)	<0.001
CAS with multiple partners	559 (30.8)	194 (20.3)	0.57 (0.47 to 0.69)	<0.001	0.55 (0.45 to 0.67)	<0.001
Unmet need for STI testing†	611 (42.5)	314 (32.8)	0.66 (0.55 to 0.78)	<0.001	0.58 (0.48 to 0.70)	<0.001

Table created by coauthors.

*Adjusting for age, ethnic group, country of residence, having a degree, relationship status.

†One or more new sex partners and/or multiple condomless anal sex partners, and no STI testing.

CAS, condomless anal sex; MSM, men who have sex with men.

Among participants included in the comparative analysis, 32.8% met our definition of unmet need for STI testing in 2020 compared with 42.8% in 2017. In multivariable analysis, participants in 2020 were less likely to have unmet need (aOR=0.58, (0.48 to 0.70), p<0.001).

## Discussion

We found considerable potential for BBSTI transmission during the first national lockdown (March–July 2020) in the UK, with 37% of participants reporting one or more new partners and 17% reporting multiple CAS partners. The combined data on sexual risk behaviour and STI testing indicated that one quarter of participants had unmet need for this type of sexual healthcare during lockdown. We found less unmet need for STI testing during lockdown compared with 2017 but inequalities in access to sexual healthcare during lockdown among black and Asian participants, those living in Scotland or Northern Ireland, and people living with HIV. Use of SHS before and after lockdown began was commensurate with changes in service provision, with online self-sampling for BBSTI testing much more likely to be reported during lockdown, alongside a reduction in service attendance for routine, asymptomatic check-ups. We found a reduction in sexual activity among MSM during lockdown compared with our 2017 survey.

There is inconsistent evidence on the impact of the social distancing measures introduced due to the COVID-19 pandemic on sexual behaviour among MSM (online supplemental appendix 6). 17–21 However, our finding of a reduction in sexual activity with continued risk for HIV/STI transmission has been found elsewhere.^7 8 17–19^ Other aspects of what may be described as COVID-19 ‘sexual distancing’^22^ include increased use of phone sex, web cams and pornography,^8 18 20^ and in our study, about one-sixth of participants had engaged in virtual sex since lockdown began.

Modelling research has indicated that a decrease in sexual partnerships and interruption of clinical services over 18 months would reduce STI transmission.^22^ Our analysis also suggested a reduction in unmet need for STI testing during lockdown compared with 2017. The shift from face-to-face testing to self-sampling is in line with findings of a rapid uptake in HIV home-testing among US MSM during social distancing.^23^

There is, however, concern that those at greatest risk of poor sexual health may be the worst affected by the pandemic.^24^ The adverse impacts of financial hardship on MSM are highlighted,^19 25^ and reduced access to HIV prevention tools has been reported in the USA^25^ and globally,^24^ as well as discontinued use of PEP and PrEP in the UK.^7 9^ We observed inequalities in access to STI testing among minority ethnic participants and people living with HIV. Our data suggest a discontinuation of PrEP use among some MSM and commensurate reduction in risk behaviour since lockdown began, but we cannot disentangle whether decreased access to PrEP is driving changes in sexual behaviour or vice versa.

While our definition of unmet need for STI testing is normative and pragmatic, it should be noted that it may both overestimate and underestimate unmet need for some individuals. Collecting data under rapidly changing circumstances is challenging and our survey was fielded over a time when lockdown rules began to relax across all four UK nations. Compared with an MSM survey fielded earlier in the first UK national lockdown (April–May 2020),^7^ our data, collected a few months later, suggest that sexual risk behaviour may have increased during this time. We cannot, however, determine whether the behaviours reported in our survey’s 3-month lookback period occurred before or after the relaxation of rules across the UK in early July 2020.

As with most community-based surveys of MSM, our findings are based on a convenience sample, limiting the potential to generalise to the population as a whole. However, the participants were recruited widely from social media and dating apps to reach a broad sample and the repeat design provides some confidence in the equivalence of samples for tracking changes over time. Data were collected from across the UK and the distribution across the four nations is broadly equivalent to the distribution of the general population.^26^

Compared to our 2017 survey, we broadened the eligibility criteria to include gender-diverse people and collect data from groups that have been under-represented in sexual health research.^12^ We did not, however, recruit enough participants to explore these groups in any detail. As with all cross-sectional surveys, the data do not provide evidence of a causal link between sexual behaviour and service use.

This study provides vital intelligence to support public health messaging, future public health planning and efforts to mitigate against risks to health during and after the COVID-19 response,^27^ including potential inequalities in access to sexual healthcare among minority ethnic people. The findings will inform the interpretation of routine surveillance data over the course of the pandemic by providing insight on underlying sexual health behaviour and need. They will aid preparation for a potential ‘rebound’ in sexual risk behaviour when social distancing measures end, any rapid increase in infection transmission, outbreaks and pressure on services, including provision for those who may have delayed seeking healthcare.

The data reported here are from the first of three waves of cross-sectional surveys that will track sexual behaviour, service use and unmet need across time. Further work is needed to explore intersectionality and how social restrictions may undermine personal well-being and mental health among groups experiencing discrimination or disadvantage. Future research is also needed to understand the sexual health needs of transgender and gender-diverse people in the UK, and to explore whether meeting sex partners from outside one’s household has impacted transmission of COVID-19.

**Key messages**

- Reduced service provision in the 3 months following the first UK national lockdown was not associated with increased unmet need for STI testing among men who have sex with men (MSM).
- MSM in the UK continued to report sexual behaviour during this time, putting them at risk for HIV and STI transmission.
- Potential inequalities in access to STI testing during lockdown were found among black and Asian MSM and those living with HIV.

## Data Availability

Data are available upon reasonable request.
