# Supplementary material for: ‘Stay at home …’: exploring the impact of the COVID-19 public health response on sexual behaviour and health service use among men who have sex with men: findings from a large online survey in the UK
Source: Sex Transm Infect. 2021 Sep 20:sextrans-2021-055039. doi: 10.1136/sextrans-2021-055039 (PMC8457994; doi:10.1136/sextrans-2021-055039)
Supplement: Supplementary file 1 [file sextrans-2021-055039supp001.pdf]

Appendix 1 – Programme of research

These surveys were conducted as part of the RiiSH programme of research (Reducing inequalities and improving Sexual Health). The first RiiSH online MSM survey was conducted in 2017 [14,15] and the present survey is the first of three planned rapid response surveys, designed to capture comparative data during the COVID-19 pandemic. The design of the 2020 survey was based on the 2017 survey.

Howarth AR, *et al. Sex Transm Infect* 2021;0:1–7. doi: 10.1136/sextrans-2021-055039

Appendix 2 – Lookback periods for 2017 and 2020 surveys

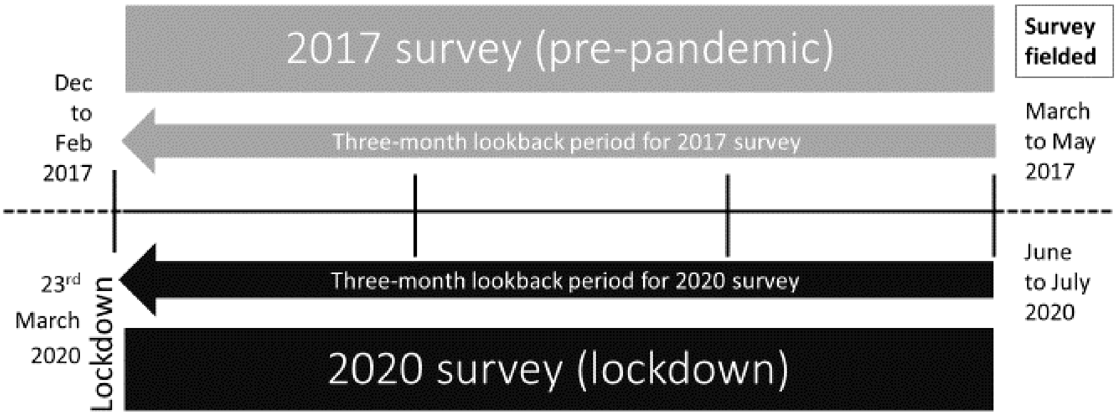

Figure created by co-authors

Howarth AR, *et al. Sex Transm Infect* 2021;0:1–7. doi: 10.1136/sextrans-2021-055039

Appendix 3 – Background characteristics, by recruitment site

	All – n (%) Median [IQR] N=2018	Social media – n (%) Median [IQR] N=950	Dating apps – n (%) Median [IQR] N=1068	p-value
**Cisgender man**	1958 (97.0)	919 (96.7)	1039 (97.3)	.47
**Age (in years)**	40 [29–52]	41 [29-53]	40 [30-50]	.13
**Ethnic group**				.001
White	1781 (88.3)	862 (90.7)	919 (86.0)	
Black	33 (1.6)	8 (0.8)	25 (2.3)	
Asian	109 (5.4)	37 (3.9)	72 (6.7)	
Mixed / Other	95 (4.7)	43 (4.5)	52 (4.9)	
**Country of residence**				.44
England	1744 (86.4)	827 (87.1)	917 (85.9)	
Scotland	144 (7.1)	63 (6.6)	81 (7.6)	
Wales	85 (4.2)	43 (4.5)	42 (3.9)	
Northern Ireland	45 (2.2)	17 (1.8)	28 (2.6)	
**Born in the UK**	1577 (78.1)	753 (79.3)	824 (77.2)	.25
**Educated to degree level**	1174 (58.2)	563 (59.3)	611 (57.3)	.36
**Employment status**				.06
Employed / self-employed	1165 (58.1)	533 (56.2)	632 (59.8)	
Furlough, reduced hours, redundancy	407 (20.3)	183 (19.3)	224 (21.2)	
Student	116 (5.8)	63 (6.6)	53 (5.0)	
Unemployed	142 (7.1)	77 (8.1)	65 (6.1)	
Retired / other	176 (8.8)	93 (9.8)	83 (7.9)	
**Last sex with a women [§]**				.35
Since lockdown	71 (3.5)	29 (3.1)	42 (3.9)	
December 2019 to lockdown	16 (0.8)	4 (0.4)	12 (1.1)	
January to December 2019	35 (1.7)	16 (1.7)	19 (1.8)	
Before 2019	332 (16.5)	157 (16.5)	175 (16.4)	
Never	1564 (77.5)	744 (78.3)	820 (76.8)	
**Household**				
Living alone	722 (35.8)	305 (32.1)	417 (39.0)	.001
Living with partner(s)	614 (30.4)	382 (40.2)	232 (21.7)	<.001
**Currently single**	1192 (59.1)	428 (45.1)	764 (71.5)	<.001
**HIV positive**	203 (10.1)	98 (10.3)	105 (9.8)	.72

[§] Including trans women

Table created by co-authors

Howarth AR, *et al. Sex Transm Infect* 2021;0:1–7. doi: 10.1136/sextrans-2021-055039

Appendix 4 – Unmet need for STI testing[±] since lockdown, by English region of residence

	Unmet need n (%)	p-value
All England	148 (24.0)	
**Region**		.53
East Midlands	27 (26.7)	
East of England	38 (27.3)	
London	132 (22.4)	
North East	13 (22.8)	
North West	49 (28.8)	
South East	60 (21.8)	
South West	37 (27.6)	
West Midlands	30 (23.4)	
Yorkshire and Humber	24 (18.8)	
Don’t know	6 (27.3)	

± One or more new sex partners and / or multiple condomless anal sex partners, and no STI testing

Table created by co-authors

Appendix 5 – Comparable background characteristics, by survey

	All – N=2874	2017 survey – N=1918	2020 survey – N=956	p-value
	**Median [IQR]**	**Median [IQR]**	**Median [IQR]**	
**Age (in years)**	38 [29-48]	37 [28-48]	40 [30-50]	<.001
	**n (%)**	**n (%)**	**n (%)**	
**Ethnic group**				.02
White	2524 (88.4)	1700 (89.5)	824 (86.2)	
Black	64 (2.2)	43 (2.3)	21 (2.2)	
Asian	147 (5.1)	82 (4.3)	65 (6.8)	
Mixed / Other	121 (4.2)	75 (3.9)	46 (4.8)	
**Country of residence**				<.001
England	2581 (90.6)	1774 (93.7)	807 (84.4)	
Scotland	92 (3.2)	12 (0.6)	80 (8.4)	
Wales	147 (5.2)	106 (5.6)	41 (4.3)	
Northern Ireland	30 (1.1)	2 (0.1)	28 (2.9)	
**Born in the UK**	2251 (79.0)	1506 (79.6)	745 (77.9)	.31
**Educated to degree level**	1525 (53.3)	965 (50.7)	560 (58.6)	<.001
**Currently single**	1869 (65.1)	1191 (62.3)	678 (70.9)	<.001
**HIV positive**	319 (11.1)	223 (11.7)	96 (10.0)	.18

Table created by co-authors

Howarth AR, *et al. Sex Transm Infect* 2021;0:1–7. doi: 10.1136/sextrans-2021-055039

Appendix 6 – Evidence on the impact of the social distancing measures introduced due to the COVID-19 pandemic on sexual behaviour among MSM

Nine out of ten gay and bisexual men (GBM) in a study in the United States (US) reported one or no sexual partners during this time;[18] in a Belgian study, the proportion of MSM reporting sex with casual partners decreased from 59% to 9%;[19] and an Australian study found only 16% of GBM continued having sex with casual partners.[17] By comparison, two fifths of MSM in an Israeli study continued meeting new casual sex partners, albeit fewer in number,[20] and high levels of sexual activity have been reported in another US study, along with increases in substance use and binge drinking.[21]
